# Decreased blood antioxidant capacity and increased lipid peroxidation in young cigarette smokers compared to nonsmokers: Impact of dietary intake

**DOI:** 10.1186/1475-2891-6-39

**Published:** 2007-11-08

**Authors:** Richard J Bloomer

**Affiliations:** 1 Department of Health and Sport Sciences, The University of Memphis, Memphis, TN, USA

## Abstract

**Background:**

Blood of cigarette smokers routinely displays decreased antioxidant capacity and increased oxidized lipids compared to nonsmokers. This is thought to be due to both chronic exposure to cigarette smoke in addition to low intake of dietary antioxidants, and is a routine finding in veteran smokers. No study to date has determined the independent and combined impact of dietary intake and cigarette smoking on blood antioxidant capacity and oxidative stress in a sample of young, novice smokers.

**Methods:**

We compared resting plasma antioxidant reducing capacity (ARC; expressed in uric acid equivalents), serum trolox-equivalent antioxidant capacity (TEAC), whole blood total glutathione, plasma malondialdehyde (MDA), and plasma oxidized low density lipoprotein (oxLDL) between 15 young (24 ± 4 years), novice smokers (pack-year history: 3 ± 2) and 13 nonsmokers of similar age (24 ± 5 years). Detailed dietary records were maintained during a seven-day period for analysis of total energy, macro- and micronutrient intake.

**Results:**

ARC (0.0676 ± 0.0352 vs. 0.1257 ± 0.0542 mmol·L^-1^; mean ± SD, p = 0.019), TEAC (0.721 ± 0.120 vs. 0.765 ± 0.130 mmol·L^-1^, p = 0.24) and glutathione (835 ± 143 vs. 898 ± 168 μmol·L^-1^, p = 0.28) were lower in smokers compared to nonsmokers, with only the former being statistically significant. MDA (0.919 ± 0.32 vs. 0.647 ± 0.16 μmol·L^-1^, p = 0.05) and oxLDL were both higher in smokers compared to nonsmokers (229 ± 94 vs. 110 ± 62 ng·mL^-1^, p = 0.12), although only the MDA comparison was of statistical significance. Interestingly, these findings existed despite no differences in dietary intake, including antioxidant micronutrient consumption, between both smokers and nonsmokers.

**Conclusion:**

These data, with specificity to young, novice cigarette smokers, underscore the importance of smoking abstinence. Future studies with larger sample sizes, inclusive of smokers of different ages and smoking histories, are needed to extend these findings.

## Introduction

Production of reactive oxygen species (ROS) in quantities that overwhelm the endogenous antioxidant defense system is referred to as oxidative stress and involves the oxidation of molecules in ways that impair cellular function. Chronic oxidative stress has a strong association with numerous disease states including cardiovascular disease (CVD), with several excellent reviews of associated mechanisms relating oxidative stress with CVD recently presented [1-4]. Cigarette smokers have an increased risk of CVD, possibly mediated by elevated levels of oxidized macromolecules owing to heightened ROS production.

Smokers are exposed to significant quantities of ROS in both the gas and tar phase [5]. Further ROS production mediated through inflammatory processes may exacerbate those produced through direct exposure [6]. Previous investigations indicate that smokers have elevated resting biomarkers of oxidative stress compared to nonsmokers [7-9]. Subjects in these studies have traditionally been older, more established smokers, with significant pack-year histories (>20).

Potential explanations for the elevated levels of oxidative stress biomarkers in a population of smokers include both increased ROS production from smoke exposure as well as an impaired antioxidant defense system. While cigarette smokers often have lower blood levels of antioxidants compared to nonsmokers, it remains unclear whether or not this occurs primarily as a function of decreased dietary intake of antioxidant rich foods [10-13] or depletion of circulating antioxidants through chronic smoke exposure [14,15]. The few studies that controlled for dietary levels of isolated antioxidant nutrients as reviewed by Alberg [14] have suggested that smoking may independently lead to selective depletion in blood levels of the nutrient of focus. Unfortunately, these investigations have not considered "total" antioxidant capacity, rather focusing on vitamin C or the carotenoids, while many investigations have simply failed to control for dietary factors altogether. Moreover, while blood antioxidant capacity is important, the oxidation of molecules that may contribute to disease, such as low density lipoprotein (LDL), appears most important. Lastly, in the one study that has measured multiple antioxidant vitamins in relation to smoking and dietary intake [15], subjects were older (mean age of 43 years) and more established smokers (mean pack-year history of 27). Therefore, the purpose of this investigation was to determine the independent and combined effect of cigarette smoke exposure and dietary antioxidant intake on blood antioxidant capacity and lipid peroxidation, and to do so in a population of young, novice smokers. It was found that young, novice smokers (pack-year history of 3 ± 4) have lower blood antioxidant capacity and greater lipid peroxidation compared to nonsmokers, despite having similar dietary intake.

## Methods

### Participants

Fifteen cigarette smokers and 13 nonsmokers volunteered to participate in this investigation. During an initial screening visit, participants completed a health history questionnaire and underwent a physical examination. All participants were sedentary and free of major signs and symptoms suggestive of cardiovascular and pulmonary disease. No participant used nutritional supplements (e.g., antioxidants) or medications (e.g., anti-inflammatory or cardiovascular drugs) that may have affected the dependent variables being measured. Participants needed to regularly smoke ≥5 cigarettes per day to be enrolled as a smoker and needed to be smoking continuously for a minimum of six months prior to being enrolled. Nonsmokers were those participants who had never smoked based on self report, and were not routinely exposed to environmental tobacco smoke in social situations. All experimental procedures were in compliance with the Helsinki Declaration and approved by the University Human Subjects Review Board and participants provided both verbal and written consent prior to participating. Participant characteristics are presented in Table 1.

**Table 1:** Characteristics of smokers and nonsmokers.

Variable	Smokers (n = 15)	Nonsmokers (n = 13)
Age (yrs)	24 ± 4	24 ± 5
Height (cm)	173 ± 8	172 ± 8
Weight (kg)	81 ± 14	77 ± 15
BMI (kg·m^-2^)	26.7 ± 4	25.5 ± 3
Resting Heart Rate (bpm)	70 ± 3	67 ± 3
Resting SBP (mmHg)	120 ± 12	116 ± 10
Resting DBP (mmHg)	78 ± 10	74 ± 10
Cigarettes per day	11 ± 10	NA
Years smoking	6 ± 5	NA
Pack-Years	3 ± 4	NA

Note: Values are mean ± SD. No statistical differences were noted between groups for any of the above variables (p > 0.05). BMI, body mass index; SBP, systolic blood pressure; DBP, diastolic blood pressure.

### Blood collection and biochemistry

Approximately one week following the initial screening visit, participants reported to the laboratory in a fasted state. Smokers were instructed to refrain from smoking for the one hour period prior to reporting to the lab as previously suggested by Dietrich et al. [15]. Venous blood samples were collected via needle and vacutainer following a 10-minute quiet rest period. A portion of whole blood was deproteinated using 5% metaphosphoric acid and then assayed for total glutathione using commercially available reagents (Northwest Life Science Specialties, Vancouver, WA). The remainder of whole blood was separated immediately to plasma or serum and stored in multiple aliquots at -80°C. Plasma antioxidant reducing capacity (ARC; represented as uric acid equivalents) was determined as a measure of blood antioxidant capacity using commercially available reagents (Northwest Life Science Specialties, Vancouver, WA). Serum trolox-equivalent antioxidant capacity (TEAC) was determined using procedures outlined by the reagent provided (Sigma Chemical, St. Louis, MO). Malondialdehyde (MDA) was analyzed in plasma using the method described by Jentzsch et al. [16]. Plasma oxidized LDL (oxLDL) was determined using an enzyme linked immunosorbent assay (ELISA) procedure (Alpco Diagnostics, Salem, NH). Both MDA and oxLDL were used as markers of lipid peroxidation. Assays were performed in duplicate on first thaw.

### Dietary records

In order to compare dietary intake between smokers and nonsmokers, detailed diet records were maintained by all participants for the seven days prior to providing their blood sample. During the period of recording, participants were instructed to record all food and drink consumed, and were strongly encouraged to consume their normal diets. Using standardized procedures for dietary data collection that our unit has used for several years [17,18], participants were provided with written instructions on how to complete the food records in addition to personal instruction during their screening visit. Upon return of records, each entry was reviewed with participants to assure accuracy. Although the necessity for thorough descriptions by the participant and detailed review by the researchers has been questioned [19], we chose to continue with this protocol until more definitive data appear. These procedures, as well as dietary data entry and management, were overseen by the same research assistant. Records were analyzed for total kilocalories, protein, carbohydrate, fat, vitamin C, vitamin E, vitamin A, B vitamins, and selected minerals using commercially available software (Diet Analysis Plus, ESHA Research, Salem, OR). This analysis allowed for nutrient intake assessment between smokers and nonsmokers.

### Statistical analysis

All data were compared between smokers and nonsmokers using a one-way ANOVA. Effect size calculations for all bloodborne variables were performed using Cohen's D. Regression analysis for the each dependent variable (e.g., glutathione, ARC, TEAC, MDA, and oxLDL) was performed using the following predictor variables: smoking status (smoker/nonsmoker), vitamin C, vitamin A, vitamin E. These antioxidant nutrients were chosen as they have been reported previously to be impacted by cigarette smoke, as well as alter levels of oxidative stress and antioxidant biomarkers. The distribution of data for each predictor was checked for normality prior to inclusion. Pairwise correlations were performed on all predictor variables in order to determine multi-collinearity. If correlations above 0.90 were observed between predictor variables, only the variable with the higher R^2 ^values was included. None of the predictor variables needed to be excluded for this reason. Following these steps, block regression using smoking status and dietary antioxidant intake (vitamin C, vitamin A, vitamin E; as one block) was performed. In a separate analysis, smoking history based on the number of cigarettes smoked per day and the number of years smoked were included as independent predictors of the bloodborne variables. All analyses were performed using JMP statistical software version 4.0 (SAS Institute, Cary, NC). Statistical significance was set at p < 0.05. The data are presented as mean ± SD.

## Results

Antioxidant reducing capacity was lower (p = 0.019) and MDA was higher in smokers compared to nonsmokers (p = 0.05). Total glutathione (p = 0.28) and TEAC (p = 0.24) were not statistically different between smokers and nonsmokers, although were both slightly lower for smokers. Results for oxLDL approached statistical significance (p = 0.12) and oxLDL was more than two-fold higher in smokers compared to nonsmokers. Effect size calculations for ARC, MDA, and oxLDL were considered to be moderate. Data for all bloodborne variables are presented in Table 2.

**Table 2:** Bloodborne variables for smokers and nonsmokers.

Variable	Smokers (n = 15)	Nonsmokers (n = 13)	P value	Effect Size
Antioxidant Reducing Capacity (mmol·L^-1^)	0.0676 ± 0.0352	0.1257 ± 0.0542	0.019*	0.54
Trolox-Equivalent Antioxidant Capacity (mmol·L^-1^)	0.721 ± 0.120	0.765 ± 0.130	0.24	0.17
Total Glutathione (μmol·L^-1^)	835 ± 143	898 ± 168	0.28	0.20
Malondialdehyde (μmol·L^-1^)	0.919 ± 0.32	0.647 ± 0.16	0.05*	0.47
Oxidized Low Density Lipoprotein (ng·mL^-1^)	229 ± 94	110 ± 62	0.12	0.60

Note: Values are mean ± SD.

Dietary intake for smokers and nonsmokers was not statistically different for total kilocalories, macronutrients or micronutrients (p > 0.05, Table 3). Regression analysis indicated that the classification of smoker (smoker or nonsmoker) contributed to the greatest percentage of the variability in ARC (R^2 ^= 0.22), MDA (R^2 ^= 0.31), and oxidized LDL (R^2 ^= 0.11) when compared to individual dietary components. This was accounted for primarily by the number of years smoking as opposed to the number of cigarettes smoked per day. For example, within smokers it was noted that the number of years smoking accounted for the greatest variability in ARC (R^2 ^= 0.26) and MDA (R^2 ^= 0.22), although less variability was explained by this regressor in oxLDL (R^2 ^= 0.12), glutathione (R^2 ^= 0.04), and TEAC (R^2 ^= 0.08). The number of cigarettes smoked per day did not explain a large portion of the variability in any of the dependent variables. Multiple regression with the addition of vitamins C, A, and E (as one block) increased the R^2 ^to 0.33 for ARC, to 0.41 for MDA, and to 0.22 for oxLDL. Smoking and dietary intake contributed little to explaining the variability in total blood glutathione and TEAC.

**Table 3:** Dietary intake during the seven days prior to providing blood sample for smokers and nonsmokers.

Participants	Kcal	Protein	Carbohydrate	Fat	Vitamin C	Vitamin E	Vitamin A	Iron
Smokers	1850 ± 666	65 ± 20 (18 ± 8)	242 ± 85 (51 ± 8)	66 ± 28 (31 ± 4)	37 ± 20	4 ± 2	667 ± 387	14 ± 5
Nonsmokers	1643 ± 302	61 ± 19 (15 ± 4)	214 ± 55 (52 ± 10)	60 ± 20 (33 ± 8)	54 ± 47	5 ± 3	499 ± 548	12 ± 7

Note: Data are mean ± SD. Gram quantities for each macronutrient are provided with corresponding percentages in parentheses. Vitamin C, vitamin E, and iron values are provided in mg; vitamin A values are provided in retinol equivalents. No statistical differences were noted between groups for any measured variable shown above or for any of the B vitamins, vitamin D, calcium, sodium, potassium, phosphorous, or zinc (p > 0.05).

## Discussion

Data from the present study indicate that young, novice smokers (pack-year history of 3 ± 4) have a lower plasma antioxidant capacity and exhibit a greater degree of lipid peroxidation compared to nonsmokers, despite having similar dietary intake. These data suggest that the act of cigarette smoking may independently promote such negative changes, with the number of years spent smoking contributing most to these findings. While dietary intake, as well as other genetic and environmental factors not determined in this investigation may contribute to these results, it appears that smoking plays a major role in promoting these changes.

It is known that exogenous antioxidant intake from whole food and nutritional supplements may influence both the antioxidant capacity of blood [20] as well as oxidative stress biomarkers [21]. It has been independently reported that smokers consume less antioxidant rich foods compared to nonsmokers [11-13] and have suppressed blood levels of certain antioxidants such as ascorbic acid [15], tocopherol, and superoxide dismutase [22], which may influence the degree of oxidative stress. As such, many investigators have reported elevated levels of oxidative stress biomarkers in smokers compared to nonsmokers [7-9]. However, no previous study has determined the independent and combined contribution of smoking and dietary antioxidant intake on blood antioxidant capacity and oxidative stress biomarkers, in particular within a population of young, novice smokers. Previous studies have included older, more established smokers, as in the work of Dietrich and coworkers [15] in which subjects had a mean age of 43 years and a mean pack history of 27 years. In contrast, our participants had a mean age of 24 years and a mean pack history of only 3 years. While these findings are interesting, they are highly specific to young, novice smokers. Future studies with larger sample sizes, inclusive of smokers of different ages and smoking histories, are needed to extend these findings. In this way, smokers could be classified by age, as well by smoking habit (e.g., light, moderate, and heavy). Using this approach, data would better be able to be generalized to the population at large.

We chose to measure blood antioxidant capacity as well as two common markers of lipid peroxidation, MDA and oxLDL. A great deal of focus has been placed on the oxidative modification of lipids, and in particular LDL and the causal role of oxLDL in the pathophysiology of atherosclerosis [23,24]. Oxidized LDL is more atherogenic than native LDL and is taken up by the scavenger receptor system ultimately leading to the generation of foam cells and the development of early lesions [25]. Atherosclerotic lesions in both animal and man have been reported to contain significant oxLDL [26], while antibodies to oxLDL have been found to correlate with the progression of atherosclerosis [27]. Oxidized LDL is also cytotoxic and has the ability to promote endothelial dysfunction, as well as the induction of genes such as interleukin-1 that can induce smooth muscle cell proliferation and promote a procoagulant state [28]. Furthermore, oxLDL may promote platelet adhesion, trigger DNA strand breaks, and promote apoptosis, all of which contribute to the development of atherosclerotic disease [29]. Based on the above, we believe that oxLDL is an important marker to focus on in relation to oxidative stress research. Although we failed to note statistical significance between smokers and nonsmokers with regards to oxLDL, values were more than two-fold higher in smokers and our effect size calculation for this marker was moderate. It is likely that we were underpowered statistically to detect significance in oxLDL. Future studies with larger samples are needed to corroborate our findings.

It should be noted that despite no differences in dietary variables between smokers and nonsmokers, mean vitamin C, vitamin E, and vitamin A intake was lower than the recommended Dietary Reference Intakes for both groups of participants. The current recommended daily intake of vitamin C is 75 mg per day for women and 90 mg per day for men 19–50 years of age. Daily vitamin E intake is suggested at 15 mg per day for both men and women, while vitamin A intake is suggested at 700 μg per day for women and 900 μg per day for men 19–50 years of age. It is possible that the lower than recommended intake of these vitamins could have promoted a lower antioxidant capacity and higher lipid peroxidation. However, because we found no statistical difference between groups for these variables, we have no reason to believe that one group was affected more than another in this regard. While participants were instructed to record all food and drink consumed during the reporting period, it is possible that underreporting could have occurred. If so, the analyzed values may have been lower than what participants habitually consume. This is indeed a limitation of the present investigation and of using dietary records within a free living environment to determine nutrient intake.

## Conclusion

These findings indicate that young, novice cigarette smokers have lower blood antioxidant capacity and higher lipid peroxidation levels compared to nonsmokers, despite having similar dietary intake. This is the first report to suggest that the act of cigarette smoking, in particular the number of years participating in this activity, may manifest in impaired antioxidant capacity and elevated oxidative stress independent of dietary intake. In particular, these data are in reference to young, novice smokers. It is very possible that more robust findings in relation to nutrient intake would be observed in a population of older, more established smokers. Future study is needed to confirm this hypothesis. Based on previous literature, such changes over time appear to have the potential to promote ill-health and disease within susceptible individuals. Additional studies using larger samples with the inclusion of clinically relevant endpoints are needed to extend these findings.

## Competing interests

The author(s) declare that they have no competing interests.

## Authors' contributions

R. Bloomer was responsible for the study design, oversight of all data collection, biochemical work, statistical analyses, and manuscript preparation.
